# A hospital outbreak of seasonal influenza involving three health care workers – implications on the optimal choice of respiratory protection

**DOI:** 10.1186/1753-6561-5-S6-P100

**Published:** 2011-06-29

**Authors:** B Wong, R Lai, P Chan, N Lee

**Affiliations:** 1 Medicine and Therapeutics, Prince of Wales Hospital, Hong Kong, Hong Kong, China; 2 Microbiology, Prince of Wales Hospital, Hong Kong, Hong Kong, China

## Introduction / objectives

The guidance on the optimal choice of respiratory protection for health care workers (HCWs) caring for patients with suspected/confirmed influenza infection requiring non-invasive ventilation (NIV) has remained controversial.

## Methods

We investigated an influenza A outbreak involving 3 HCWs in a medical ward in July 2009. Contact tracing & viral studies were performed to identify the source of outbreak.

## Results

Three HCWs (a nurse, an intern & a general services assistant (GSA)) reported fever and upper respiratory symptoms on July 13. Nasal/throat swabs confirmed influenza A infection. On contact tracing, all 3 reported contact history with 2 patients (Patient A & B) admitted to the ward on July 10 & 11 respectively who were later diagnosed with influenza A. Patient A was on 2L/min O_2_ while Patient B developed respiratory failure requiring 100% O_2_ and was soon transferred to a side room and put on NIV. Virological investigations confirmed that Patient B & the 3 staffs had influenza A/H3 subtype infection, while Patient A had influenza A/H1 infection.

The nurse had cared for both patients, the intern had performed physical examination for both patients, and the GSA transported both patients to the isolation facilities. They reported good compliance to droplet precautions & hand hygiene and were wearing surgical mask but not mucosal protection during the care of both patients. These 3 were the only staff among 24 that reported working within close proximity (<1 meter) of Patient B while he was on NIV.

## Conclusion

Patient B was identified as the index case causing this outbreak of influenza A/H3 subtype.

Droplet precaution and the use of surgical mask may not offer adequate respiratory protection to HCWs caring for patients on NIV.

## Disclosure of interest

None declared.

